# Imaging Regional Lung Structure and Function in Small Animals Using Synchrotron Radiation Phase-Contrast and K-Edge Subtraction Computed Tomography

**DOI:** 10.3389/fphys.2022.825433

**Published:** 2022-03-08

**Authors:** Sam Bayat, Luca Fardin, José Luis Cercos-Pita, Gaetano Perchiazzi, Alberto Bravin

**Affiliations:** ^1^Univ. Grenoble Alpes, Inserm UA07 STROBE Laboratory, University of Grenoble Alpes, Grenoble, France; ^2^Department of Pulmonology and Clinical Physiology, Grenoble University Hospital, Grenoble, France; ^3^European Synchrotron Radiation Facility, Grenoble, France; ^4^Hedenstierna Laboratory, Department of Surgical Sciences, Uppsala University, Uppsala, Sweden; ^5^Department of Physics, University of Milano-Bicocca, Milan, Italy

**Keywords:** pulmonary function, synchrotrons, computed tomography, respiration artificial, regional blood flow

## Abstract

Synchrotron radiation offers unique properties of coherence, utilized in phase-contrast imaging, and high flux as well as a wide energy spectrum which allow the selection of very narrow energy bands of radiation, used in K-edge subtraction imaging (KES) imaging. These properties extend X-ray computed tomography (CT) capabilities to quantitatively assess lung morphology, and to map regional lung ventilation, perfusion, inflammation, aerosol particle distribution and biomechanical properties, with microscopic spatial resolution. Four-dimensional imaging, allows the investigation of the dynamics of regional lung functional parameters simultaneously with structural deformation of the lung as a function of time. These techniques have proven to be very useful for revealing the regional differences in both lung structure and function which is crucial for better understanding of disease mechanisms as well as for evaluating treatment in small animal models of lung diseases. Here, synchrotron radiation imaging methods are described and examples of their application to the study of disease mechanisms in preclinical animal models are presented.

## Introduction

An ideal technique for imaging regional lung function should provide both high spatial and temporal resolution, allow for quantitative measurements of functional parameters and provide the ability to image the underlying lung morphology. Structural and functional imaging data along with computational modeling have significantly contributed to our understanding that lung function as a whole cannot be predicted by the sum of the behavior of individual components, but results rather from the interaction of components at multiple scales ranging from biomolecular and cellular to different lung regions, which leads to complex dynamic phenomena such as self-organization and emergence (Suki and Bates, 2011). Human, animal and cell culture studies have demonstrated that mechanical strain on the lung tissue and alveoli, plays a crucial role in processes such as lung growth and repair, surfactant release and inflammation (Roan and Waters, 2011). However, capturing lung structure and function simultaneously at small length scales remains a very technically challenging goal *in vivo*, and despite advances in both imaging technology and understanding of lung mechanics over the past decades, still little is known about lung micromechanics and how lung alveoli and acini deform during breathing (Roan and Waters, 2011; Smaldone and Mitzner, 2012).

Conventional X-ray imaging with laboratory or clinical sources, is based on absorption contrast. A multitude of interesting methods and algorithms have been developed for conventional micro-CT of the lung, with prospective and retrospective gating, motion compensation, and radiation dose reduction *via* sophisticated reconstruction algorithms. These have been extensively reviewed previously (Ashton et al., 2015; Clark and Badea, 2021). However, conventional CT is limited by the low radiation flux available in standard X-ray imaging systems, which reduces the spatial and temporal resolution, particularly in *in vivo* imaging.

Synchrotron radiation on the other hand, offers unique properties of high flux, wide energy spectrum and coherence, meaning that the photons are to a large degree spatially and temporally in phase. The high flux as well as a wide energy spectrum allow the selection of very narrow energy bands of radiation, utilized in K-edge subtraction imaging (KES) imaging. The coherence of the radiation is utilized instead in phase-contrast imaging. Here, the distortion of the X-ray wave front in the lung leads to strong edge enhancement within the images due to interference of the transmitted and refracted radiation. In weakly attenuating tissues such as the lung, refraction can be orders of magnitude greater than absorption, particularly at higher energies. The propagation-based imaging technique (PBI) has proven to be especially suited for lung imaging due to sharp edge enhancement caused by the air-to-tissue interfaces in the lung microstructures (4, 5).

Four-dimensional (4D) CT imaging, in which high-resolution mapping of lung functional parameters is recorded simultaneously with structural deformation of tissue morphology as a function of time, provides the basis for comprehensive modeling of the dynamics of lung function, at spatial resolutions allowing the visualization of alveoli. Recently, impressive results toward this goal have been achieved using synchrotron radiation sources (Cercos-Pita et al., DOI: 10.21203/rs.3.rs-970496/v, *under review*).

The physical instrumentation and optical methods of these imaging techniques has been reviewed in detail recently (Bayat et al., 2020). In this mini-review, methodological aspects of KES-CT and propagation-based 4D-CT lung microscopy are summarized.

## Animal Preparation

**FIGURE 1:**
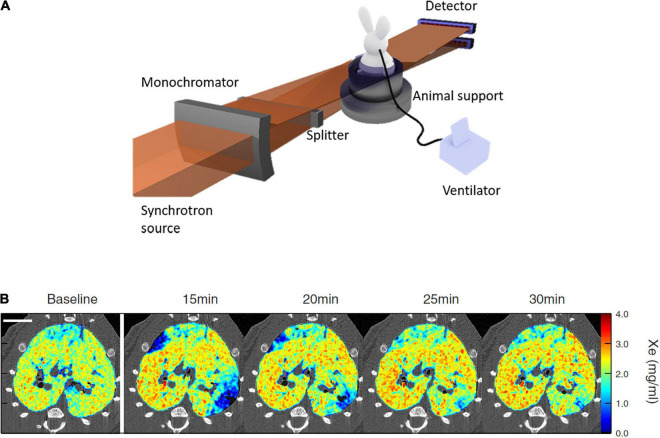
**(A)** Schematic of synchrotron radiation K-edge subtraction imaging (KES) setup; **(B)** sample composite images showing the Xe concentration distribution repeatedly imaged by KES with 47 μm voxel size, in ovalbumin-sensitized Brown-Norway Rats challenged with inhaled ovalbumin. Scale bar represents 10 mm. Note the transient emergence of regional lung ventilation defects after ovalbumin challenge. Reproduced with permission from Layachi et al. (2013), Bayat et al. (2021).

*In vivo* imaging with synchrotron radiation requires dedicated instrumentation and remote control. The synchrotron beam is a stationary horizontal fan so that the animal is subjected to rotation up to 180–360°/s as well as vertical displacement for 2D-projection imaging. While it is possible to acquire images during free breathing, obtaining high resolution maps of regional ventilation using this imaging method usually requires the respiration to be controlled by mechanical ventilation while the physiological parameters are continuously monitored and recorded. Other global measurements of cardiovascular (e.g., ECG, invasive blood pressure) and respiratory function such as respiratory mechanics (Bayat et al., 2009) or inert gas multiple breath washout (Bayat et al., 2013) can be performed in parallel to image acquisition using the experimental setup (Figure 1). Mechanical ventilation can be ensured using *ad hoc* systems allowing synchronization with the image acquisition or commercial and even clinical mechanical ventilators (Porra et al., 2016), as long as these devices can be remotely controlled for example to pause respiration in inspiration or expiration for imaging. Imaging the lung in static conditions in apnea typically requires pauses of 1–3 s in lower-resolution KES imaging and 10–60 s in higher resolution phase-contrast imaging. Longer apnea durations in small animals require preoxygenation followed by apneic oxygenation by high-flow O_2_ at the airway opening, in order to avoid O_2_ desaturation during imaging (Frumin et al., 1959).

Anesthesia is usually induced by intramuscular injection of a mixture of ketamine and xylazine (rabbit, rat, mouse), or IV injection of thiopental sodium *via* a catheter (22 G) introduced into the marginal ear vein under local anesthesia (5% topical lidocaine) in rabbits. Anesthesia is then maintained by IV infusion (rabbit, rat) or IP injection (mouse) of a ketamine and xylazine mixture. Anesthesia can also be maintained by inhaled volatile anesthetics. Controlled mechanical ventilation can be delivered through tracheal intubation or most often tracheostomy with a polyethylene tracheal tube. For some imaging protocols, after ensuring adequate depth of anesthesia, muscle relaxation is induced by continuous IV infusion of atracurium (rabbit) or IP pancuronium bromide (rat, mouse). Depth of anesthesia is monitored by regularly assessing the state of the pupils (rabbit) and heart rate. Because the radiation beam is horizontal, the animal is typically placed in a custom-made plastic holder for imaging in upright position (Porra et al., 2004). However, local tomography can also be performed in supine position in small animals (unpublished data).

Physiological parameters, such as the ECG, respiratory pressure and flow, arterial pressure, oxygen saturation, among other parameters, can be monitored using an analog/digital interface. This allows not only to monitor and record such parameters in order to assess the physiological condition and welfare of the animal, but also to collect scientifically important data such as respiratory mechanics and hemodynamic parameters. Parameters such as the ECG and respiratory pressures also allow to trigger image acquisition in a prospectively synchronized fashion with cardiac and respiratory function (Fardin et al., 2021). Also, the inhaled fraction of O_2_ can be set and inert or tracer gas administration switched remotely by the image acquisition software or manually (Bayat et al., 2021) in order to image regional lung ventilation.

## K-Edge Subtraction Computed Tomography Functional Lung Imaging

The K-edge subtraction computed tomography (KES-CT) technique allows simultaneous imaging of the lung tissue morphology, and the concentration (mass per unit of volume) of inhaled Xenon gas within the airspaces. The instrumental setup for this imaging modality has been reviewed in detail previously (Bayat et al., 2020). This imaging technique uses two monochromatic X-ray beams at slightly different energies bracketing the K-edge of inhaled Xe (34.56 keV) for ventilation imaging, or injected iodine (33.17 keV) for blood volume and perfusion imaging. Visualization and quantitative measurement of contrast concentration in the lung is based on the property that the attenuation coefficient of a contrast element increases severalfold when the energy of the incident X-ray beam exceeds the K-edge of that element. X-rays from a synchrotron radiation source are required because, as opposed to standard X-ray sources, they allow the selection of monochromatic beams from the full X-ray spectrum while conserving enough intensity for imaging with sufficient temporal resolution. KES-CT imaging is performed in parallel-beam geometry. Two CT images are simultaneously acquired during the Xe inhalation maneuver, using a solid state (Bayat et al., 2021) or a charge-coupled device (CCD) detector (Layachi et al., 2013; Figure 1A). The size of the field of view is determined by that of the radiation beam and the detector resolution. For example, in a recent study (Bayat et al., 2021) the horizontal beams were 98 mm wide and 0.6 mm in height, and focused on a rabbit. In that study, each CT image consisted in 720 projections over 360° per 1.5 s. CT images were reconstructed using a filtered back projection algorithm with resulting voxel dimensions of 350 μm × 350 μm × 700 μm. At this voxel size, the distribution of xenon gas within lung acini could be assessed in rabbits, in order to investigate down to which length scale ventilation remains inhomogeneous in normal lungs. Using fractal analysis, it was demonstrated that ventilation becomes internally uniform within regions about the size of rabbit lung acini (∼5 mm^3^) (Bayat et al., 2021).

At a higher voxel resolution of 47 μm^2^, the acquisition time is longer (∼10 s) (Figure 1B). Using the dual-energy KES synchrotron imaging method, X-ray attenuation by tissue density and Xe concentration is computed separately, using a custom material decomposition algorithm as described previously (Bayat et al., 2001). At this resolution, Layachi et al. (2013) found higher eosinophil, monocyte and total cell densities within vs. outside lung regions where ventilation defects emerged following allergen inhalation in ovalbumin-sensitized Brown-Norway rats.

K-edge subtraction computed tomography image acquisition can be performed dynamically during a single (Bayat et al., 2021) or in-between multiple inspirations or expirations (Bayat et al., 2013). The resulting images can be used to compute a map of regional ventilation based on the regional time-constant of Xenon washin or washout. In the case of Xenon washin, as the alveoli are gradually filled by the gas (Porra et al., 2004):


$$C_{t}=C_{a\,s}\,\left[1-e^{-\left(t-t_{0}\right)\,\tau }\right]$$

where C_*t*_ is the gas concentration as function of time, C_*as*_ the asymptotic concentration and τ the time constant. As ventilation within a lung region increases, the time constant of regional Xe washin or washout becomes shorter. Specific ventilation ($s\,\dot{V}$), or ventilation per unit of regional gas volume, is defined by:


$$s\,\dot{V}=\frac{1}{\tau }$$

A similar approach can be used to compute maps of regional blood volume and perfusion (Suhonen et al., 2008).

The spatial resolution and contrast sensitivity of KES imaging is mainly determined by the characteristics of the detection system. For example, in studies performed using a solid-state cooled germanium detector, a pixel size of 0.33^2^ mm^2^ and a sensitivity better than 0.1 mg/ml could be obtained. Smaller pixel sizes of 47 μm^2^ have previously been achieved in *in vivo* KES-CT imaging using a charge coupled device (CCD) detector (Layachi et al., 2013). This makes KES a unique method because of the high spatial resolution and absolute scale of the contrast element distributions.

## Phase-Contrast Functional Lung Imaging

The lung poorly attenuates X-rays. However, the numerous air-tissue interfaces within the lung airways and alveoli result in refraction and phase changes of the incident X-rays. Phase-contrast X-ray imaging (PCI), uses the phase information in addition to attenuation to enhance contrast within poorly-attenuating structures (Bravin et al., 2013). This technique takes advantage of the high degree of spatial coherence provided by synchrotron X-ray sources. In addition to improving contrast, this imaging approach has the advantage of reducing the radiation dose in comparison to conventional X-ray attenuation imaging (Lewis et al., 2005). The numerous air-tissue interfaces crossed by the incident beam within the lung produce phase gradient patterns which resemble random noise or “speckles,” unlike the surrounding soft tissues (Kitchen et al., 2004).

**FIGURE 2:**
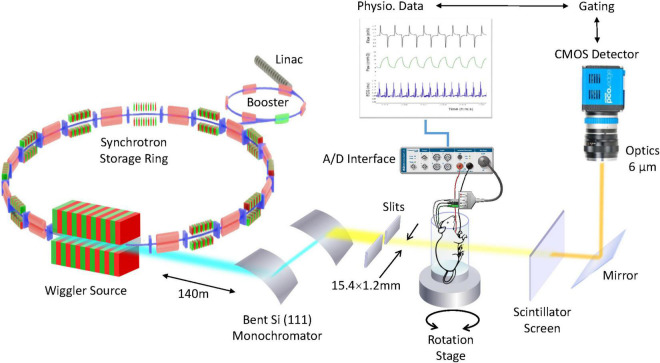
4D microscopy of rat lung using a synchrotron X-ray source. High-intensity coherent X-rays generated from electrons orbiting in a storage ring, are rendered monochromatic using bent silicon crystal optics, and detected by a PCO Edge 5.5 camera coupled to a Cerium-doped Lutetium Aluminum Garnet (LuAG:Ce) scintillator and optics yielding an isotropic pixel size of 6 μm^3^. The *in vivo* anesthetized rat is mechanically ventilated while the electrocardiogram and respiration are monitored and recorded. Reproduced from (Cercos-Pita et al., doi: 10.21203/rs.3.rs-970496/v1, *under review*).

Propagation-Based Imaging (PBI), is the simplest and most widely used method because no X-ray optical devices are needed (Figure 2). In this configuration a small X-ray source provides a high spatial coherence, an essential condition to visualize the phase effects. Another essential condition is a sufficient distance between the object and the detector, which is chosen as a function of the X-ray energy and the detector pixel size. There are several methods for phase retrieval from the observed intensity distribution, which also includes the effects of absorption (Nesterets et al., 2015).

Examples of the application of this imaging technique to study regional lung deformation and function are discussed in a recent review (Bayat et al., 2021). *In vivo* phase-contrast synchrotron radiation tomography allows the measurement of regional lung aeration with high contrast sensitivity, short acquisition times compatible with *in vivo* imaging, and the ability to acquire 3D data at sub acinar spatial resolution. However, the spatial resolution of static imaging approaches is limited by blurring due to cardiac and vascular motion, which hinders the study of aeration and deformation within individual alveoli.

This issue is addressed by 4DCT imaging. This approach involves gating the acquisition of individual image projections with the cardiac and respiratory motions. Moreover, the exposure time of each image projection needs to be reduced as the microscopic features of interest become smaller, which makes 4DCT microscopy technically challenging (Bayat et al., 2018). The high photon flux and coherence of synchrotron radiation are necessary for this technique, as well as accurate physiologic signal acquisition and control software allowing precise triggering of all components of the image acquisition process. Resolving alveolar structure in the lung is particularly challenging due to inhomogeneous speed and magnitude of the physiological tissue motion. Acquiring abundant tomographic data can help resolve this issue.

Under static lung inflation conditions, Lovric et al. (2017) studied lung inflation patterns during diastole at the alveolar scale *in vivo*, with a voxel size of 2.9^3^ μm^3^, in anesthetized 9-day old rats. They acquired 450 individual projections at 3 ms exposure time, for a total acquisition time of 2 min (Lovric et al., 2017). Their data demonstrate the feasibility of eliminating motion artifacts due to cardiac activity and resolving alveolar structure *in vivo*. Using a prospective cardiac gating technique, the authors were able to image mouse lungs at 1.1 μm voxel size during static breath hold conditions (Lovric et al., 2017). However, imaging the lung in static conditions is less physiological and does not allow capturing the full scope of local lung mechanics. This is because the lung tissue is viscoelastic, meaning that its apparent elastic properties depend on the rate of volume change (Suki and Bates, 2011). Dynamic imaging techniques are therefore needed to map lung biomechanics, ideally at spatial resolutions allowing to resolve the pulmonary alveoli.

**FIGURE 3:**
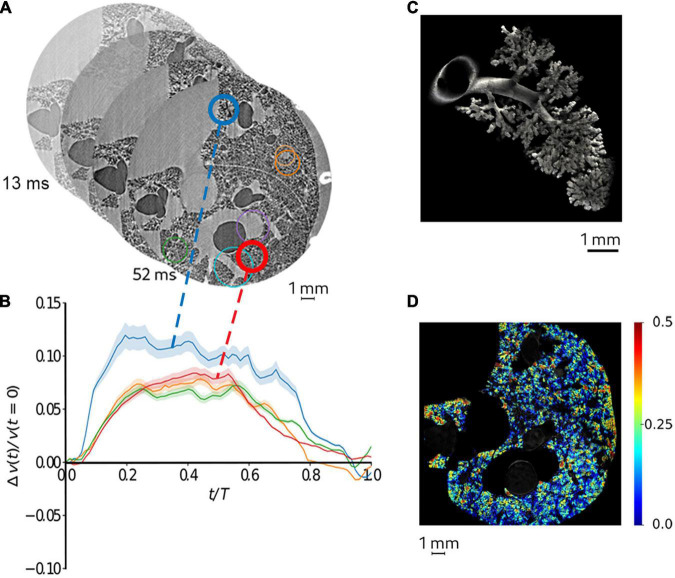
Quantitative mapping of lung tissue biomechanics in a live rat. **(A)** sample sequential X-ray phase-contrast CT images at successive time points, reconstructed by retrospectively sorting of 250,000 individual 2 ms image projections with respect to the phase of heart contraction and breathing, yielding 78 time points during the breath; **(B)** regional strain as a function of time computed within airspaces in the regions of interest of same color as in panel **(A)**. The shaded area represents within-ROI standard deviation; **(C)** a segmented airway with subtending conducting airways and terminal acinar structures at end-expiration in a live rat; **(D)** sample regional strain map of airspaces *in vivo* in the same animal. Color bars indicate strain (*δV/V_*t0*_*, where *t*_0_ is the start of the breath). Reproduced from (Cercos-Pita et al., doi: 10.21203/rs.3.rs-970496/v1, *under review*).

Recently, Cercos et al. investigated lung tissue deformation induced by cardiac contractions and respiration in anesthetized adult rats, showing the magnitude and regional inhomogeneity in this deformation in intact *in vivo* lungs (Cercos-Pita et al., DOI: 10.21203/rs.3.rs-970496/v, *under review*). By synchronizing image acquisition with both respiration and cardiac activity, 250,000 projections with 2 ms integration time were retrospectively acquired over 180° in 8.8 min. Both the respiratory and cardiac-induced motion could be resolved using this technique in mechanically ventilated live rats, at 6 μm^3^, and 78 time points during a breath (Figure 3). This study shows that 4D tomographic microscopy is a valuable technique not only for assessing local lung structure but also for quantitatively mapping local biomechanics at microscopic length scales. The main limitations of the technique are the length of data acquisition ranging up to several minutes, and the risk of excessive radiation dose which can alter the underlying tissue structures. Also, dynamic imaging limits the spatial resolution due to motion blurring induced by breathing in addition to cardiovascular motion. More sensitive detection devices and specifically designed imaging end stations can help mitigate these limitations in the future.

## Future Perspectives and Challenges

Real-time imaging of lung function is highly challenging, particularly *in vivo* due to motion blurring and the non-linear deformation of the lung tissue with breathing and cardiovascular motion. There is a trade-off between spatial and temporal resolutions, and both are difficult to achieve simultaneously. *In vivo* synchrotron radiation micro-CT also faces limitations due to radiation dose, and a limited field of view. However, in KES-CT, limitations due to radiation exposure can be overcome by reducing the number of projections and using iterative reconstruction algorithms while maintaining sufficient contrast resolution for quantitative mapping of ventilation (Strengell et al., 2014), while PCI has the advantage of reducing the radiation dose in comparison to conventional X-ray attenuation imaging (Lewis et al., 2005; Zhao et al., 2012). The radiation beam produced by a synchrotron source is stationary, which imposes translation and rotation of the sample through the beam for image acquisition. Another challenge posed by fast acquisition 3D imaging is handling the large volume of data, which can rapidly represent several terabytes. Large data volumes cannot be visualized in real time with conventional approaches. A change in the data representation paradigm, from the classical Cartesian grid to a hierarchical data structure is therefore mandatory to allow a real-time visualization on different planes as well as morphological analysis in a reasonable time. This in turn, requires adapting image processing algorithms. Synchrotrons are large research infrastructures that are not widely available. However, several facilities worldwide are accessible to the scientific community through a competitive peer-reviewed process based on scientific merit (Quitmann and Rayment, 2020).

A limitation of KES-CT is a contrast sensitivity that is far smaller than fluorescence or radionuclide imaging. However, an exciting development is the ability to track high atomic number nanoparticles loaded within cells (Schültke et al., 2014; Hubert et al., 2021), or functionalized in order to reveal a specific molecular target. This would allow taking functional imaging utilizing synchrotron radiation a step further toward molecular imaging. Possibilities to achieve these challenging goals exist at current synchrotron facilities with recent progress in detection, acquisition and data processing capabilities.

## Author Contributions

SB wrote the first draft of the manuscript. LF, JC-P, GP, and AB wrote sections of the manuscript. All authors contributed to manuscript revision, read, and approved the submitted version.

## Conflict of Interest

The authors declare that the research was conducted in the absence of any commercial or financial relationships that could be construed as a potential conflict of interest.

## Publisher’s Note

All claims expressed in this article are solely those of the authors and do not necessarily represent those of their affiliated organizations, or those of the publisher, the editors and the reviewers. Any product that may be evaluated in this article, or claim that may be made by its manufacturer, is not guaranteed or endorsed by the publisher.
